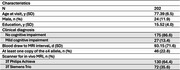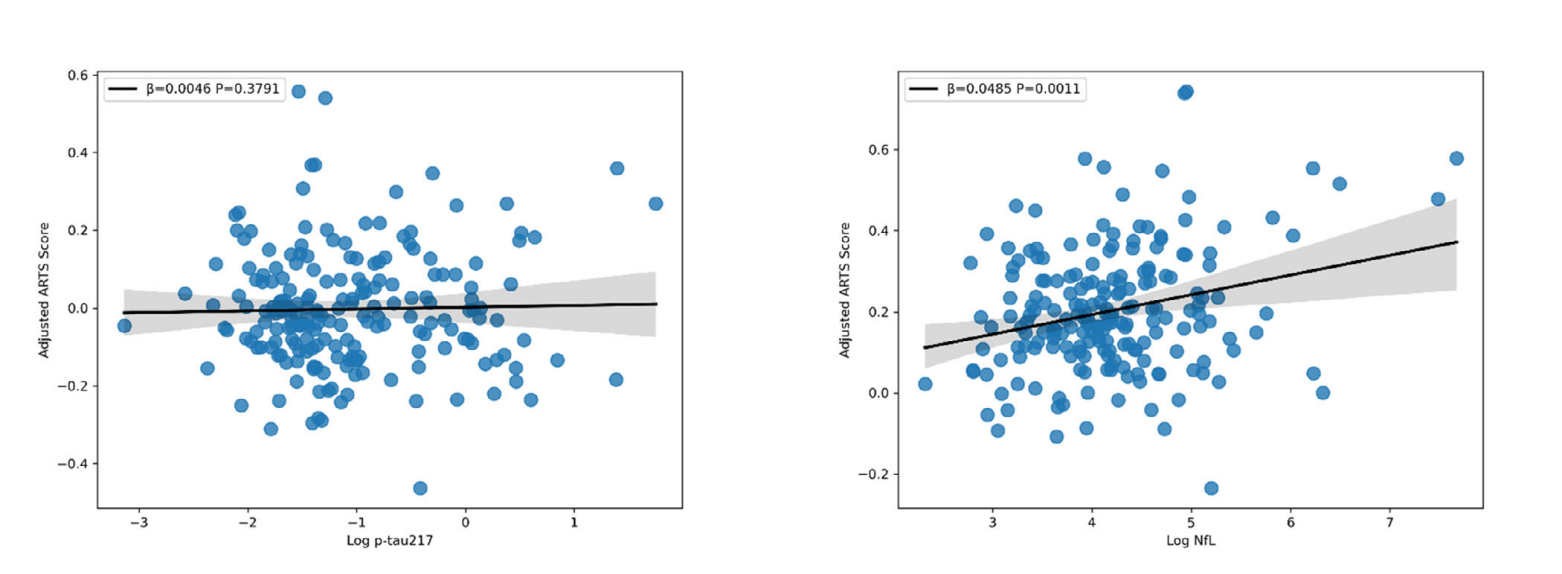# ARTS is associated with NfL but not with *p*‐tau217

**DOI:** 10.1002/alz70856_101643

**Published:** 2025-12-24

**Authors:** Gulam Mahfuz Chowdhury, Khalid Saifullah, Arnold M Evia, David A. A. Bennett, Julie A Schneider, Konstantinos Arfanakis

**Affiliations:** ^1^ Illinois Institute of Technology, Chicago, IL, USA; ^2^ Rush Alzheimer's Disease Center, Chicago, IL, USA; ^3^ Rush Alzheimer's Disease Center, Rush University Medical Center, Chicago, IL, USA

## Abstract

**Background:**

Intracranial arteriolosclerosis, one of the main pathologies of cerebral small vessel disease (CSVD), has a significant impact on cerebrovascular health and cognition. ARTS is a recent MRI‐based marker of arteriolosclerosis, and is the first to provide in‐vivo insight about the presence of arteriolosclerosis pathology. Phosphorylated tau 217 (*p*‐tau217) is a blood‐based biomarker for AD pathology, and plasma neurofilament light chain (NfL) is a biomarker of both neurodegenerative‐related and vascular‐related axonal damage. In this work, we investigated the association of ARTS with blood‐based biomarkers *p*‐tau217 and NfL.

**Method:**

This study included 202 participants from four longitudinal, clinical‐pathologic cohort studies of aging: MAP, ROS, MARS, and ADRC including only participants with no or mild cognitive impairment.

All participants underwent in‐vivo MRI on 3 Tesla MRI scanners using MPRAGE, FLAIR, and DTI sequences. The MRI data were used to calculate ARTS scores for all participants using the fully‐automated, publicly available ARTS software. Plasma *p*‐tau217 and NfL levels were measured at NCRAD using established procedures.

Linear regression tested the association of ARTS score with *p*‐tau217 and NfL levels, separately, controlling for demographics (age at visit, sex, years of education), interval between blood draw and MRI scanning, and scanner (Figure 1). The FSL PALM tool with 10000 permutations and tail acceleration was used for the statistical analysis. Associations were considered significant at *p* <0.05 after family‐wise error (FWE) correction.

**Result:**

No significant association of ARTS scores with levels of *p*‐tau217 was detected (β=0.0046, *p* = 0.38), underscoring that ARTS is independent of Alzheimer's pathology (Figure 2A). In contrast, a strong positive association of ARTS with NfL was observed (β=0.0485, *p* = 0.0011), highlighting that ARTS is linked to CSVD‐related axonal injury (Figure 2B).

**Conclusion:**

The present study in community‐based older adults without dementia suggests that ARTS scores are not influenced by Alzheimer's pathology, which often co‐occurs with arteriolosclerosis. Yet, higher ARTS scores are associated with more severe axonal injury assessed by means of higher NfL scores. Both findings are in support of ARTS as a specific marker of arteriolosclerosis and cerebral small vessel disease.